# Genetics Selection Evolution reviewer acknowledgement 2013

**DOI:** 10.1186/1297-9686-46-1

**Published:** 2014-01-28

**Authors:** Didier Boichard, Jack CM Dekkers, Helene Hayes

**Affiliations:** 1 INRA, UMR 1313 Génétique Animale et Biologie Intégrative, 78350, Jouy-en-Josas, France; 2 Department of Animal Science and Center for Integrated Animal Genomics, Iowa State University, Ames, IA, USA

## Abstract

**Contributing reviewers:**

The *Genetics Selection Evolution* Editors-in-Chief would like to thank all of our reviewers who contributed to peer review for the journal in 2013.

## 

Behnam Abasht

United States of America

Syuiti Abe

Japan

Samuel E Aggrey

United States of America

Ignacio Aguilar

Uruguay

Panoraia Alexandri

Netherlands

Peter Amer

New Zealand

Benoit Auvray

New Zealand

Jörn Bennewitz

Germany

Peer Berg

Denmark

Patrick Berrebi

France

Donagh Berry

Ireland

Francois Besnier

Norway

Jean-Pierre Bidanel

France

Piter Bijma

Netherlands

Stephen Bishop

United Kingdom

Yuna Blum

France

Paul Boettcher

Italy

Aniek Bouwman

Netherlands

Henk Bovenhuis

Netherlands

Gudrun Brockmann

Germany

Rasmus Froberg Brøndum

Denmark

Albert Johannes Buitenhuis

Denmark

Warren Burggren

United States of America

Armando Caballero

Spain

Mario Calus

Netherlands

Javier Cañon

Spain

Rodolfo Juan Carlos Cantet

Argentina

Fernando Cardoso

Brazil

Ian Carr

United Kingdom

Alessio Cecchinato

Italy

Stanislav Cepica

Czech Republic

Isabel Cervantes

Spain

Ole F. Christensen

Denmark

Samuel Adam Clark

Australia

John Cole

United States of America

Wouter Coppieters

Belgium

Ron Crump

United Kingdom

Ino Curik

Croatia

Hans D. Daetwyler

Australia

Ingrid David

France

Dirk-Jan de Koning

Sweden

Gustavo de los Campos

United States of America

Jared Decker

United States of America

Olivier Demeure

France

Johann Detilleux

Belgium

Jerry Dodgson

United States of America

Sonja Dominik

Australia

Tom Druet

Belgium

Alain Ducos

France

Ian Dunn

United Kingdom

Klaus Ehrhardt

Germany

Jeffrey Endelman

United States of America

Malena Erbe

Germany

Jesús Fernández

Spain

W. Freddy Fikse

Sweden

Laurence Flori

France

Luca Fontanesi

Italy

Alexis Fostier

France

Luis Alberto García Cortés

Spain

Dorian Garrick

United States of America

Mathieu Gautier

France

Helene Gilbert

France

Elisabetta Giuffra

France

Michael Goddard

Australia

Felix Goyache

Spain

Christine Große-Brinkhaus

Germany

Francois Guillaume

France

Bernt Guldbrandtsen

Denmark

Anne Haberland

Germany

Satoshi Hamaguchi

Japan

Jianlin Han

Kenya

Bevin Harris

New Zealand

Rachel Hawken

United States of America

Ben Hayes

Australia

John Henshall

Australia

John Hickey

Australia

Emmeline Hill

Ireland

William Hill

United Kingdom

Yu Huang

United States of America

Laura Iacolina

Italy

Noelia Ibáñez Escriche

Spain

Florence Jaffrezic

France

Steven Janssens

Belgium

Stephen Kachman

United States of America

Pete Kaiser

United Kingdom

Kathryn Kemper

Australia

Denis Laloë

France

Susan Lamont

United States of America

Tosso Leeb

Switzerland

Timothy Leeds

United States of America

Andres Legarra

France

Johannes Lenstra

Netherlands

Jenni Leppänen

Finland

Bingshan Li

United States of America

Hui Li

China

Meng-Hua Li

China

Marie Lillehammer

Norway

Ehud Lipkij

Israel

Michael MacNeil

United States of America

Christian Maltecca

United States of America

Nathalie Mandonnet

France

Esa Mantysaari

Finland

Annette McCoy

United States of America

Molly McCue

United States of America

John McEwan

New Zealand

Theo Meuwissen

Norway

Christopher Moran

Australia

Raphael Mrode

United Kingdom

Han Mulder

Netherlands

Eduard Muráni

Germany

Daniel Natt

Sweden

Frank Nicholas

Australia

David Notter

United States of America

Jorgen Odegard

Norway

Hubert Pausch

Germany

Trinidad Perez

Spain

Miguel Perez-Enciso

Spain

Marie-Hélène Pinard-van der Laan

France

Ricardo Pong-Wong

United Kingdom

Raul Walter Ponzoni

Malaysia

Laercio R Porto-Neto

Australia

Denis Réale

Canada

Norbert Reinsch

Germany

Anne Ricard

France

Euan Rodger

New Zealand

Xavier Rognon

France

Gary Rohrer

United States of America

Suzanne Rowe

New Zealand

Mahdi Saatchi

United States of America

Radek Sanda

Czech Republic

Larry Schaeffer

Canada

Flavio Schenkel

Canada

Chris Schrooten

Netherlands

Warren Snelling

United States of America

Johann Sölkner

Austria

Anna K Sonesson

Norway

Jiuzhou Song

United States of America

Anders Christian Sørensen

Denmark

Peter Sørensen

Denmark

Juan Steibel

United States of America

Ismo Stranden

Finland

Guosheng Su

Denmark

Chuanyu Sun

United States of America

Hermann Swalve

Germany

Akarapong Swatdipong

Thailand

Jens Tetens

Germany

Robin Thompson

United Kingdom

Bruce Tier

Australia

Michèle Tixier-Boichard

France

Ali Toosi

United States of America

Miguel Angel Toro

Spain

Thierry Tribout

France

Daniel Vaiman

France

Alessio Valentini

Italy

Julius van der Werf

Australia

Marc Vandeputte

France

Luis Varona

Italy

Ana Vazquez

United States of America

Roel Veerkamp

Netherlands

Matthieu Vignes

France

Johanna Vilkki

Finland

Beatriz Villanueva

Spain

Zulma Vitezica

France

Jinliang Wang

United Kingdom

Robin Wellmann

Germany

Roman Wenne

Poland

Ian White

United Kingdom

Pam Wiener

United Kingdom

Alastair Wilson

United Kingdom

Jack Windig

Netherlands

John Woolliams

United Kingdom

Zhaoxia Yu

United States of America

Huaijun Zhou

United States of America

